# Visual Guidance and Egg Collection Scheme for a Smart Poultry Robot for Free-Range Farms

**DOI:** 10.3390/s20226624

**Published:** 2020-11-19

**Authors:** Chung-Liang Chang, Bo-Xuan Xie, Chia-Hui Wang

**Affiliations:** Department of Biomechatronics Engineering, National Pingtung University of Science and Technology, Pingtung 91201, Taiwan; w380903@gmail.com (B.-X.X.); w091135652@gmail.com (C.-H.W.)

**Keywords:** free-range chickens, mobile poultry robot, egg gathering, computer vision, visual tracking

## Abstract

Free-range chicken farming allows egg-laying hens to move freely through their environment and perform their natural behavior, including laying her eggs. However, it takes time to gather these eggs manually, giving rise to high labor costs. This study proposes a smart mobile robot for poultry farms that can recognize eggs of two different colors on free-range farms. The robot can also pick up and sort eggs without damaging them. An egg feature extraction method with automatic thresholding is employed to detect both white and brown eggs, and a behavior-based navigation method is applied to allow the robot to reach the eggs while avoiding obstacles. The robot can move towards the position of each egg via visual tracking. Once the egg is within the collection area of the robot, it is gathered, sorted and stored in the tank inside the robot. Experiments are carried out in an outdoor field of size 5 m × 5 m under different climatic conditions, and the results showed that the average egg recognition rate is between 94.7% and 97.6%. The proposed mobile poultry robot is low in production cost and simple in operation. It can provide chicken farmers with automatic egg gathering on free-range farms.

## 1. Introduction

Animal welfare policies have been receiving increased amounts of attention all over the world [1]. In poultry farming, traditional battery cages prevent the hens from performing the bulk of their natural behavior, which cause them to suffer from severe disuse osteoporosis [2,3]. Even if the environment can be improved and the activities of hens in cages managed, they will be permanently deprived of the opportunity to express most of their natural repertoire of behaviors. Many countries have therefore begun to actively formulate policies and trade measures on this issue, to protect animal welfare in the poultry production system [4,5,6,7].

Following the effective promotion and formulation of such policies, many chicken farmers have used grazing, flat feeding and enriched cage feeding methods to raise chickens, in order to allow chickens to carry out a sufficient range of activities. These methods can reduce environmental pressure, avoid reductions in egg production [8], and maintain the quality of eggs at a stable level. These chicken farmers use some semi-intensive or free-range poultry house systems to facilitate the management of chickens and also comply with animal welfare policies [9]. When using flat feeding, farmers need to gather eggs outside the nest in the field, to reduce losses. These operations take up most of the chicken farmers’ working time [10]. Laying nests can indeed reduce the labor burden of farmers in collecting eggs. However, some chickens are only territorial. When the number of chickens increases, the number of nests needs to be considered and the chickens will only snatch the nests and bite each other. If egg laying is delayed for some reason, the period for pre-laying behavior will pass, and the hen will no longer be motivated to search for a nest. In these cases, the egg may be laid outside the nest while the hen goes about other activities. This can happen, for example, when dominant hens prevent subordinate hens from entering nests [11]. Furthermore, if laying hens are raised using a grazing method, the eggs are often pecked by birds or contaminated with chicken feces. Once the chicken farmer finds that a hen is laying, he or she therefore needs to pick up these eggs immediately. Although chicken farmers keep the hens indoors during the laying period in order to facilitate the management of the poultry house, during the rest of the time, the chickens are active in the outdoor area to reduce the number of floor eggs in the outdoor area. Nevertheless, it is still possible for chickens to lay eggs in outdoor areas. If chicken farmers can try their best to allow chickens to present sufficient natural behaviors, they will be more in line with animal welfare policies. Due to the gradual aging of agricultural population and shortage of labor, the labor costs paid by farmers are relatively high, which is a drawback. Egg producers are therefore likely to present a strong interest in how to efficiently collect eggs and optimize production efficiency.

One potential solution is to use robotics [12]. Mobile robot technology has been developed over many years, and has been applied to various industries [13,14]. Most types of these robots use a two-wheeled differential drive method to regulate the direction of the machine. In addition, robotic operation systems collect environmental information via different types of sensors, which allows the robot to track the target and avoid obstacles in unknown environments [15,16]. Due to the low cost and high flexibility of mobile platforms, they are suitable for use in autonomous navigation applications on flat grounds. These types of two-wheeled robots, combined with autonomous systems, are also used in agriculture [17,18,19]. A mobile robot moving both inside and outside the poultry house can not only encourage the movement of the hens and promote their health and wellbeing, but also collect eggs from the floor at the same time. In addition, the use of mobile robots can not only improve production efficiency but also reduce the number of times that the breeder needs to enter the poultry house, thereby preventing dust and pathogenic bacteria from being introduced into the environment. Vroegindeweij et al. [20] initially proposed a path-planning method that used a mobile robot to collect floor eggs in a poultry house. This method is based on a structured environment, and the results showed that the PoultryBot can reduce the need for manual egg picking. After testing a variety of path-planning methods, the authors showed that the robot can move autonomously and gather eggs [21]. Usher et al. [22] used an ultrasonic positioning method to locate their GohBot in an indoor environment, using depth data from Microsoft Kinect to design path planning and obstacle avoidance routines. Their results indicated that the GohBot can move automatically while avoiding chickens. A robot designed by Tibot Technologies (Spoutnic, TIBOT technologies, Bretagne, France) can move around a barn in a random manner. During the movement, the robot gently forces the hens to move in a continuous way, to prevent them from laying eggs randomly on the ground.

The mobile robots described above are used in indoor environments. Once the structure of its environment is confirmed, the robot can use path-planning to navigate and collect eggs.

However, in the case of a limited aviary area, once the number of chickens raised increases, the range of activities of the chickens decreases, which limits the chickens’ ability to perform natural behaviors, including nesting, perching, scratching, foraging, running, jumping, etc. Moreover, it is expensive to imitate the outdoor natural environmental conditions in the poultry house. Therefore, it is a good practice to effectively activate the outdoor space or extend the indoor field to the outdoor.

Since the outdoor environment is often a non-structural environment without regular properties, no environmental information is known by the robot system, and no environment is seen/perceived to navigate the system, identify objects or browse landmarks [23]. If the robot needs to perform a specific task to reach the target position, a map of the areas traversed must therefore be created, and the robot must implement a localization algorithm to perform target-driven navigation. Effective path-planning can save operating time and avoid inefficient navigation. Depending on whether the environmental map information is known or unknown, the navigation method can be divided into online and offline processing [24]. In addition, coverage and point-to-point path-planning methods are most commonly used in agricultural environments. Of these, the covered path-planning method is more suitable for an entire operation field, while the point-to-point path-planning is more appropriate for performing autonomous tasks for a specific number of plants [25] because the robot uses global navigation satellite systems with real-time kinematic (GNSS-RTK) to perform robot positioning and path-planning to achieve the purpose of autonomous navigation. Once the positioning signal is interfered or jammed by attackers, these intelligent robots or agricultural equipments cannot operate normally [26].

When the map needs to be created by the robot, this is done in a coordinate frame centered on the vehicle [27]. It is also possible to use a visual lens or ranging device as an external reference to construct an area map in real time from visual image and range data [28]. During the navigation process, real-time image-processing can be used to find the desired object. Once the object is found, the appropriate operation will be determined to allow for suitable navigation. Mobile robot navigation systems usually have visual detectors, which can be used in indoor or outdoor environments to detect or track objects to achieve a specific outcome [29], such as navigation guidance [30,31,32] or feature classification [33]. This image-processing technology is often used to obtain image features in order to drive the robot to make relative response actions. In order to overcome the influence of light intensity on the feature extraction process, image preprocessing technology is commonly used to suppress or filter out the complex background noise, and a morphology-based image-processing technique is then used to extract the features of the object. In addition, there are also multi-spectral systems that use artificial lighting to mitigate the disturbance caused by natural lighting conditions [34,35]. The use of deep learning or machine learning methods can also improve image classification, and this has been applied to estimate the number of livestock [36,37], the number of egg detections [38], and in other areas [39,40]. The use of these methods can avoid object detection errors or failures caused by poor lighting conditions via traditional image-processing methods. However, the training process consumes a great deal of computing resources, and hence increases the development cost of the robot system.

Since the robot can collect eggs at the same time as moving, Vroegindeweij et al. [41] exploited the retractable nature of a coil spring to clamp eggs into the spring via the spring gap. However, when the eggs are rolled into the spring, the spring spacing is extended, which indirectly increased the risk of the eggs falling out of the spring. Furthermore, the eggs can only be stored in the coil spring. When the number of eggs is increased, there is a risk of breakage if the eggs are frequently rolled.

In this study, a smart poultry robot is proposed to detect eggs, move towards them, and collect them on a free-range farm. A feature extraction method with automatic thresholding and visual tracking is used to detect eggs and control the direction of the mobile robot. At the same time, an egg collection system is employed to gather, sort and store the eggs in storage tanks.

## 2. Methodology

This section will explain the visual guidance system used by the mobile robot, and will then explain how feature extraction and an adaptive threshold technique are used to recognize two different colors of eggs on a free-range farm. Finally, a behavior-based navigation method is illustrated for the proposed mobile robot.

### 2.1. Visual Guidance of a Mobile Robot

**Figure 1:**
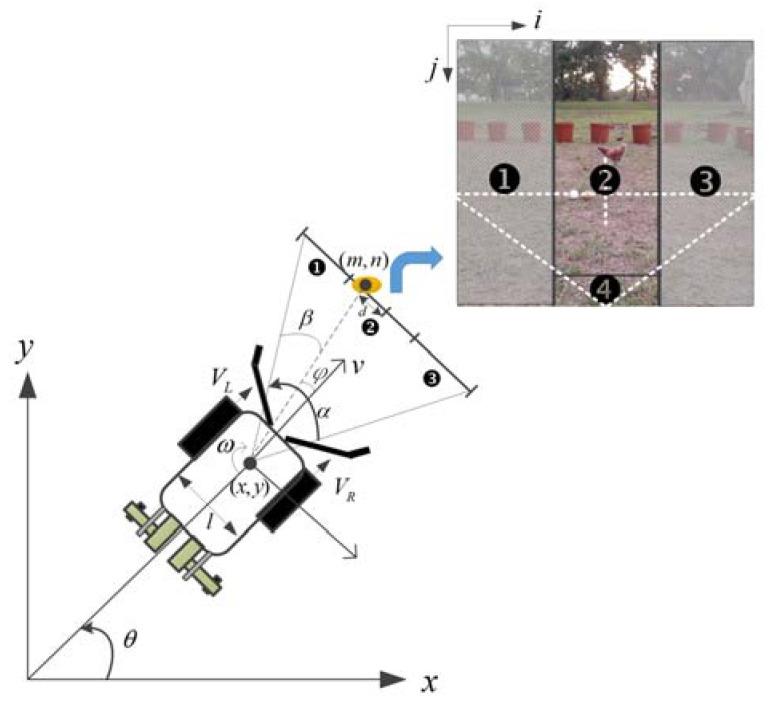
Coordinate system used for the mobile robot.

We assume that our two-wheeled steering mobile robot can move in a *x*–*y* coordinate frame (see Figure 1), and use three degrees of freedom to describe the pose: forward motion in the *x*- and *y*-axis directions, and rotation of the robot [42]. The different velocities of the right ($V_{R}$) and left ($V_{L}$) wheels result in different heading directions for the mobile robot. The forward velocity of the robot is $v=r(V_{R}+V_{L})/2$, where r represents the radius of the wheel, and $\omega =\dot{\theta }=r(V_{R}-V_{L})/2l$ is the angular velocity. The symbol l represents the distance between two wheels, and θ indicates the angle between the heading direction of the robot and the *x*-axis direction. The speeds of the right and left wheels of the robot are expressed as follows [42,43]

(1) $$\left[\begin{array}{c} V_{R}\\ V_{L} \end{array}\right]=\left[\begin{array}{cc} 1/r & l/2r\\ 1/r & -l/2r \end{array}\right]\left[\begin{array}{c} v\\ \omega \end{array}\right]$$

The image showing the area in front of the robot is divided into four action areas. When an egg is detected by the vision system, the action area in which the egg is located will be determined. When the target is recognized in action area ❷ of the image frame, the robot only needs to rotate slightly to the left or right, and then moves towards the target. In contrast, when the target appears in areas ❶ or ❸, the robot needs to quickly rotate to the left or right so that the target object enters area ❷ as soon as possible. The direction of movement of the robot depends on the steering angle *φ*: if *φ* is negative, the robot rotates clockwise; otherwise, it rotates counterclockwise. When the position of the egg is within area ❹, it is within collection range.

During navigation, if there are multiple eggs within the image plane that are classified by the vision system, the one with the smallest distance *d* between the center of the egg and the center of the image is regarded as the target. Assume the center position of the egg is (m,n) in *i–j* image coordinates, α is the angle of the field of view, and W is the width of the image frame (in pixels). The parameter β=m×(α/W) is the angle relative to the center of mass on the *i*-axis of the image frame. The velocities of the two wheels can be written as

(2) $$V_{R}=V_{max}\;V_{L}=V_{max}$$

where $V_{max}$ represents the max heading speed of the robot. If β=α/2, the robot will move straight toward the egg object in the image frame, and the desired velocity can be expressed as

(3) $$v=\frac{\eta r}{2}\left(V_{R}+V_{L}\right)=\frac{\eta rV_{max}}{2}$$

where η is a constant used to adjust the velocity of the robot.

### 2.2. Egg Recognition Process

**Figure 2:**
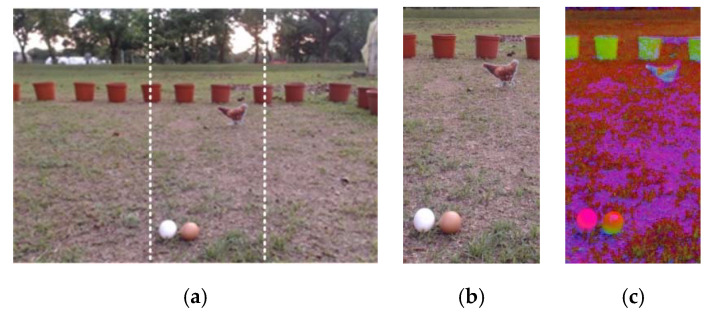
The egg recognition process: (**a**) original image; (**b**) screenshot of the central area in the image; (**c**) image after color transformation.

**Figure 3:**
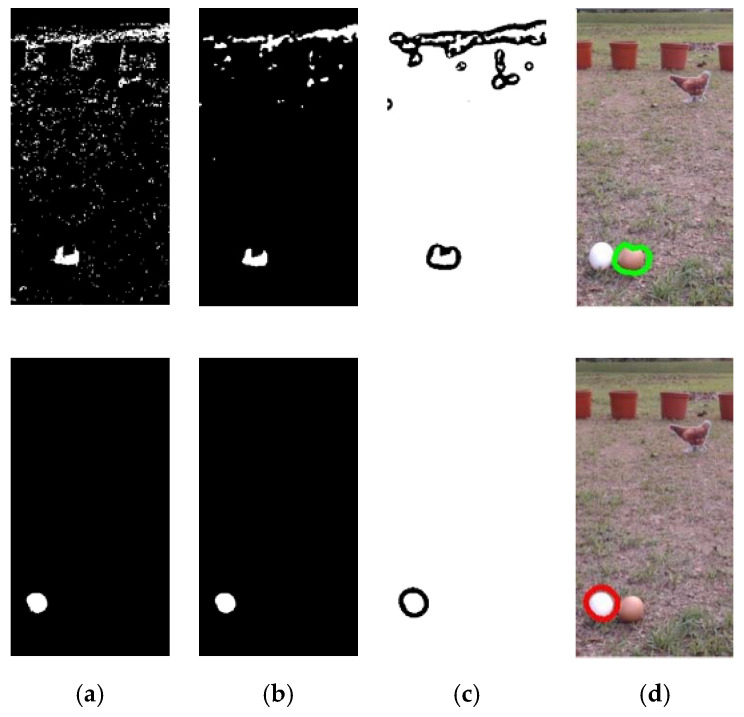
Egg recognition results for brown (top) and white eggs (bottom): (**a**) remove background; (**b**) median filtering; (**c**) contour finding; (**d**) polygon fitting and roundness test.

During the process of egg collection, the mobile robot needs to determine whether there are eggs within the image frame, and also the positions of eggs. An image-processing method is therefore used to extract eggs from the image frame based on their features, including their appearance: color and area. The digital 8-bit RGB color image captured by the digital camera is divided into three areas: the left, center, and right images (see Figure 2a). The image-processing method is employed to detect eggs in these three images. The central area of the image is shown in Figure 2b to illustrate image processing and identify the eggs. First, we convert the RGB color space of the image to HSV color space [44]. The white and brown values in the RGB color space are then defined to correspond to the range of values for each layer in the HSV color space. In the HSV layer, white is defined as H = [0, 170], S = [0, 30], V = [245, 255] and brown as H = [0, 30], S = [65, 125], V = [130, 255]. Next, a binarization process is performed to segment the background and egg objects, and the results are shown in Figure 3a. Median filtering is then used to remove the noise and the irrelevant small objects which are still visible in Figure 3a. The result is shown in Figure 3b.

A contour-finding algorithm is then used to extract the contours of all objects in the image [45], as shown in Figure 3c. In order to reduce the amount of data required in the image-processing stage to meet the needs of real-time processing, an iterative polygonal fitting algorithm is then used to extract a contour curve composed of line segments, producing a simplified curve with fewer points [46,47,48]. The idea is then to use a polygon to fit the original contour shape. The number of polygonal corner points is used to evaluate the number of image processing operations required: the lower the number of corner points, the fewer the number of pixels and the higher the distortion, and vice versa. Suppose that there are N points between the first and end points on the contour line, and each point is expressed as $P_{1}(p_{1},\;q_{1}),\;P_{2}(p_{2},\;q_{2}),\;\cdots ,\;P_{N}(p_{N},\;q_{N})$. Then, at points $P_{1}$ and $P_{N}$ in $\bar{P_{1}P_{N}}$, we define a line in the plane using the equation

(4) $$\left(q_{1}-q_{N}\right)x+\left(p_{N}-p_{1}\right)y+\left(q_{N}p_{1}-p_{N}q_{1}\right)=0$$

If we assume $a=q_{1}-q_{N}$, $b=p_{N}-p_{1}$, $c=q_{N}p_{1}-p_{N}q_{1}$, the distance $D_{n}$ (n=2, 3, …, N−1) from a corner point $P_{n}$ (n=2, 3, …, N−1) to a line $\bar{P_{1}P_{N}}$ is as follows:

(5) $$D_{n}=\frac{\left|ax_{n}+by_{n}+c\right|}{\sqrt{a^{2}+b^{2}}}\left(n=2,\;3,\;\ldots ,\;N-1\right)$$

The corner point with the maximum distance $D_{max}^{\underset{n}{{argmax}_{n}}\;n=1}$, is selected as one of the corner points of the approximate polygon. The critical distance threshold can also be set. If the distance is greater than this threshold, the point corresponding to the distance is considered a corner point of the polygon. The distance threshold is used to determine the degree of similarity of the egg shape. After trial and error, it is set to 1% of the egg contour length. When position of each corner point on the image plane has been confirmed, the area of the fitting object can be calculated.

Finally, a roundness test is used to determine whether the objects in the image are eggs, and filter out non-related objects. Equation (6) is applied in the roundness test, and represents a circle when ε=1. For the shape of an egg, the value is between 0.8 and 0.9

(6) $$\varepsilon =2\sqrt{\pi A}/L$$

where A and L indicate the contour area and perimeter, respectively. Figure 3d shows the results of image processing after the polygonal fitting algorithm and the roundness test has been applied.

### 2.3. Adaptive Threshold

To perform object feature recognition in an outdoor environment, the irrelevant background or objects are removed from the image. However, when the background is complex and the ambient light intensity is unstable, it is hard to successfully extract the desired object [49]. Although many previous studies have developed various image segmentation methods, including histogram shape-based methods [50], histogram approximation methods [51], clustering algorithms [52], and the support vector machine method [53]; these approaches consume a great deal of computing time and resources, and are not suitable for practical applications. In [54], the researchers use threshold segmentation to extract objects, which is a simple and fast method. However, the segmented objects are of the same color, and image segmentation of other colors is not involved. In addition, the threshold setting for image segmentation in this method is still manually adjusted. In this study, the trial and error method is used to find the segmentation threshold for different light intensity ranges and provides robust egg recognition performance in different weather conditions. The measurement process will be explained in Section 4.1.

### 2.4. Behavior-Based Navigation Method

**Figure 4:**
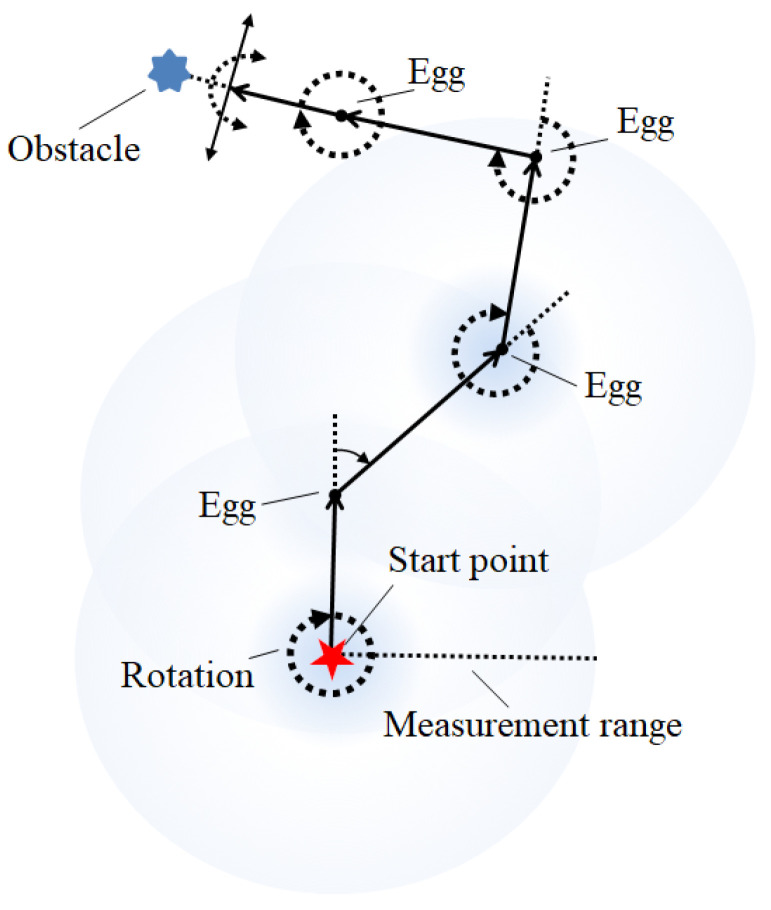
The behavior-based navigation method used for the robot.

When the robot is performing egg recognition, the robot will give priority to avoiding any obstacle it detects. In terms of the movement and behavior of the robot, a previous approach uses a walking method in which the robot first moves along the edge and then to the center of the field in a spiral manner [55]. This method is suitable for use on flat ground. However, as the robot moves toward the center, the positioning error will gradually increase, meaning that the robot’s path will not completely cover the entire field. The original path-planning method is, therefore, modified in this study to produce a simple behavior-based navigation method to steer the direction of the robot. The design concept used in the proposed method is illustrated in Figure 4. First, the robot rotates clockwise at the starting point. During this rotation, if an egg is detected, the robot will move toward it. If the robot does not find an egg after one rotation, it moves forward for a certain period of time τ.

During navigation, if an obstacle is detected in front of the robot (within a detection distance of 35 cm), it starts to rotate clockwise or counterclockwise (with the default being clockwise). During this rotation, it will detect whether there are obstacles to the left or the right. If the ultrasonic sensor on the left and right side of the robot detects an obstacle and the front ultrasonic sensor does not detect the obstacle, the robot will move forward for a certain period of time τ and then rotate again. However, if there is an obstacle in front of it, the robot will change its direction of rotation until there is no obstacle in front, and then will move forward again. If there are obstacles in front of and on both sides of the robot, it will stop.

**Figure 5:**
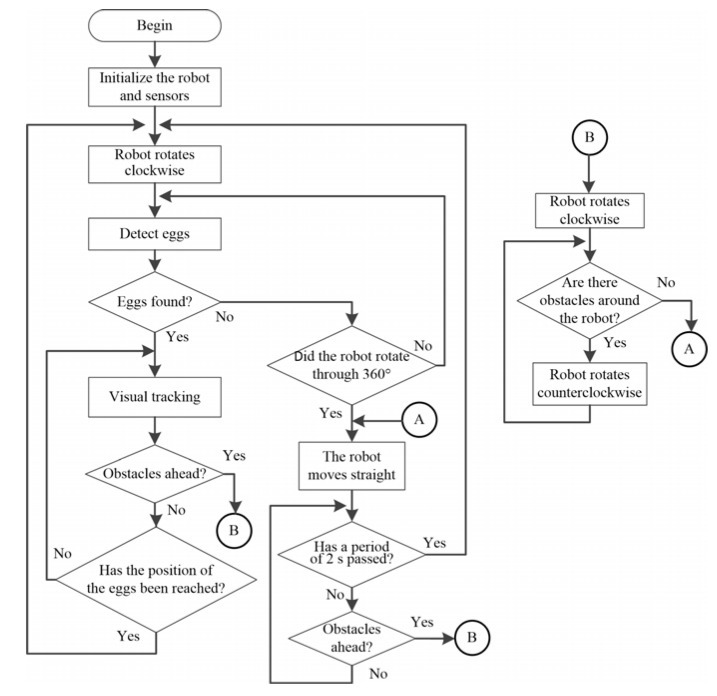
Flow chart for the behavior of the robot.

A flow chart for the operation of the robot is shown in Figure 5. During rotation, if multiple eggs are detected in the image, the robot will select the egg closest to the central area as the target, and then perform visual tracking. Equation (2) is used to obtain the rotation speeds of the left and right wheels to allow the robot to move in the direction of the egg. If the robot detects that the position of the egg is at the bottom of the image, this means that the egg has entered the robot’s collection area. If the robot keeps moving forward or rotating, the egg will be picked up by the collection device and will be placed in the egg storage tank. Hence, the distance between the robot and the egg are not measured; it is only necessary to identify the position of the egg within the plane of the image. However, the distance between the robot and an obstacle needs to be measured, and can be used to determine whether the robot needs to stop or turn to avoid an obstacle. An ultrasonic sensor is installed at the front of the robot with an effective detection distance of 35 cm. When an obstacle is detected by the robot, it rotates 90° clockwise to avoid it.

## 3. Proposed Mobile Poultry Robot

This section describes the design and mechanism of the robot, the software platform and hardware system used, and the design of the egg picking system.

### 3.1. Design of the Robot Mechanism

**Figure 6:**
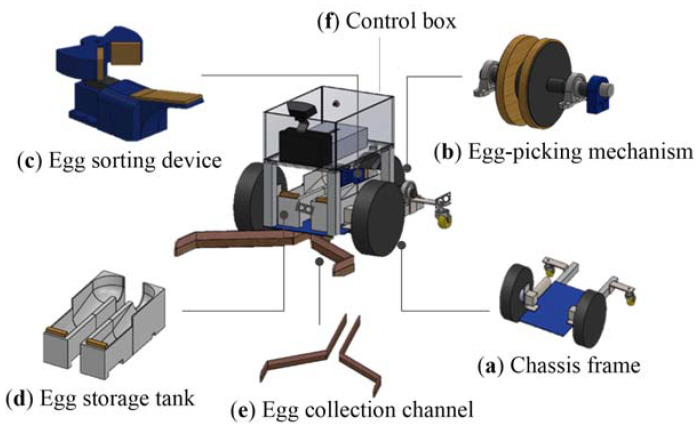
Components of the prototype robot: (**a**) classis frame; (**b**) egg-picking mechanism; (**c**) egg-sorting device; (**d**) egg storage tank; (**e**) egg collection channel; (**f**) control box.

A two-wheel speed differential drive method is used to move the robot. In a previous study [52], the egg picking and sorting mechanisms, the storage tank and the collection channel are designed. In the present work, the original egg picking mechanism, egg storage tank and drainage channel materials are modified. The mechanism of our poultry robot is illustrated in Figure 6. Figure 6a shows the robot prototype of chassis. The motor is installed on the chassis frame which is made of aluminum alloy plate. The egg-picking mechanism is demonstrated in Figure 6b. Two discs are used, and connected at the center with a cylindrical strip made of plastic steel. The inner sides of the two discs are covered with sponge to hold the collected ground eggs. The 12 V DC motor can drive the rotation of the disc at seven revolutions per minute. The egg collection and sorting mechanism consist of two fan-shaped components (see Figure 6c). A 12 V servo motor is installed at the bottom of the sorting mechanism and drives the two fan-shaped components to rotate 90° to the right or left. The purpose is to sort the eggs into the storage tanks to the right and left. The fan shape prevents two eggs from entering the sorting area at the same time. There are two egg storage tanks for brown and white eggs, and these are located on both sides of the sorting mechanism. The original egg storage tank is changed from the inclined plate structure to a circular arc slide to avoid eggs getting stuck at the entrance of the storage tank. The end of the storage tank is covered with sponge to ensure that the eggs would not be damaged as they fall. Figure 6d illustrates the prototype design of this mechanism, which can store a total of eight eggs. The structure of collection channel is similar to a funnel-shaped cavity or structure, as shown in Figure 6e. It is attached to the bottom of the chassis, and can move the eggs in front of the robot underneath it to the back to be picked up by the egg-picking carousel. The manifold is made of soft brushed plastic, which avoids the collection of irrelevant objects such as stones. The control box (see Figure 6f) is used to contain various hardware modules, drive boards and batteries, etc.

### 3.2. Hardware and Software Platforms

The size of the robot is 60 cm × 30 cm × 40 cm (length × width × height). The skeleton is made of aluminum, acrylic and plastic, and the total weight is 23 kg. The platform includes motion control and vision systems. The system specifications are shown in Table 1. The motion control system includes a microcontroller (Arduino Uno, Arduino company, Boston, MA, USA) and drive modules, which are used to drive three direct current (DC) motors and one servo motor. Of these, two DC motors (IG -42CR212-SYA123, S&J Corp., Taipei, Taiwan) are used to drive the wheels on each side of the robot, and the other (HN-35GBM-1251T, HSINEN Corp., Yuanlin City, Taiwan) is used to drive the egg-picking mechanism. The servo motor (HS-422, HITEC Inc., Chelmsford, CA, USA) is used to drive the egg-sorting mechanism. The vision system includes an embedded controller (Raspberry Pi 4 Model B, Raspberry Pi Foundation, Cambridge, UK) and two cameras. The embedded controller has a 1.5 GHz 64-bit quad-core ARM Cortex-A72 core processor and 8 GB of RAM, and can process 3–4 frames per second (FPS) for an image size of 640 × 480 pixels (width × length). Camera 1 (HD C922, Logitech Inc., Taipei, Taiwan) is installed in front of the control box and is used to capture the image in the front of the robot. Camera 2 (Raspberry Pi camera board, Raspberry Pi Foundation, Cambridge, UK) is used to capture the images of eggs, and is installed at the top of the sorting mechanism.

**Figure 7:**
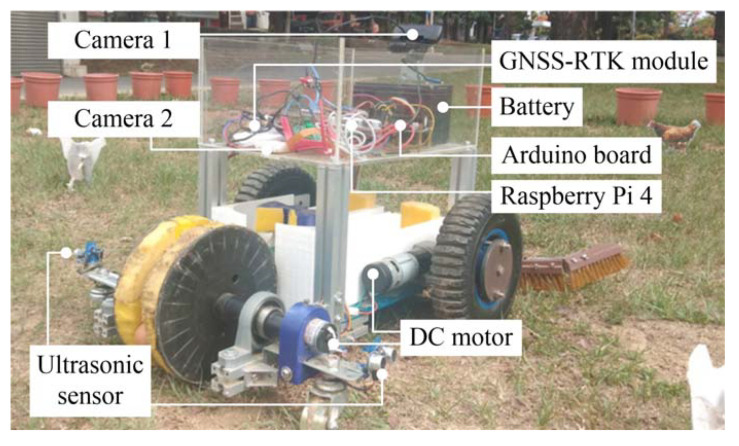
The appearance of the proposed robot.

A GNSS-RTK module (ZED-F9P, u-blox Inc., Thalwil, Switzerland) is utilized to record the navigation trajectory of the robot. Ultrasonic sensors (HC-SR04, Elec Freaks Inc., Shenzhen, China) are installed at the center of the robot to the front and at the rear on both sides to detect obstacles. The detection ranges of the front ultrasonic sensor and two rear sensors are 35 and 25 cm, respectively. The 12 V, 7.2 Ah battery capacity allows the robot to move continuously for 2.5 h. A photograph of the robot and its components is shown in Figure 7.

### 3.3. Egg Collection System

**Figure 8:**
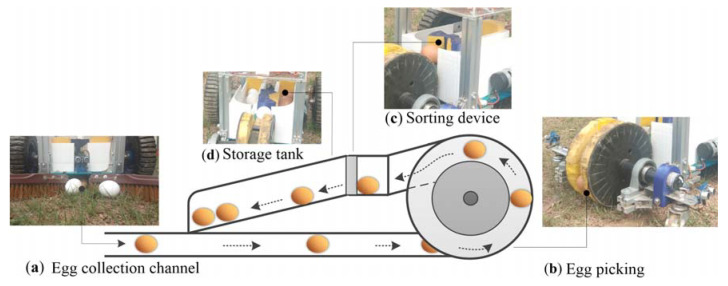
The egg collection process: (**a**) egg collection channel; (**b**) egg-picking; (**c**) sorting device; (**d**) storage tank.

In order to pick up the eggs smoothly and sort them into the storage tanks, an egg collection channel is required to gather the eggs. Figure 8 shows the egg picking and sorting process. Assume the egg is already within the egg collection range of the robot (as shown in Figure 8a), the robot continues to move forward and the eggs will enter the channel at the bottom of the robot, one at a time. Then, as the robot continues to advance, each egg will approach the turntable mechanism at the end of the robot in sequence (see Figure 8b).

The microcontroller is used to control the DC motor in the picking mechanism. As this picking mechanism rotates and the robot moves forward, the eggs are grasped by the sponge on the inside of the discs and transferred to the sorting device (see Figure 8c). Camera 1 is installed on the top of the sorting device to capture images of the eggs. The color recognition method can distinguish the color of the eggs in the image, and the method is as described in Section 2. The color recognition program is implemented in Python in the Raspberry Pi 4 embedded controller to carry out egg color recognition. When the color of the egg has been confirmed, the microcontroller executes the program instructions to drive the servo motor, and at the same time counts the number of forward and reverse rotations of the servo motor in order to calculate the number of white and brown eggs in the storage tanks. Once either storage tank (shown in Figure 8d) is filled with eggs, the picking mechanism and robot will stop.

## 4. Experimental Description and Results

This section contains an explanation of using the adaptive color threshold method to deal with the impact of unstable ambient light intensity on image recognition performance, and also analyzes the impact of the distance between the egg and the robot on image recognition performance. Finally, the eggs will be placed in the field in different configurations to actually test and evaluate the performance of the robot in picking up eggs.

### 4.1. Ambient Light Intensity Measurement

Correlations between the threshold for image binarization and the light intensity under different climatic conditions need to be established so that the vision system can regulate the threshold based on the ambient light intensity. First, the robot is set at a specific location in the field, and one white and one brown egg are placed 75 cm in front of it. The camera mounted on the robot is then used to capture the image in front of it. At the same time, the light sensor (BH1750, ROHM Co. Ltd., Kyoto, Japan) is used to record the ambient light intensity. Finally, the eggs in each photo are identified by the vision system. When eggs are detected, the binarization threshold is recorded. After applying numerical statistics to the thresholds, the threshold range of the HSV color space corresponding to different weather conditions is calculated, as shown in Table 2.

### 4.2. Initial Testing for Egg Detection

**Figure 9:**
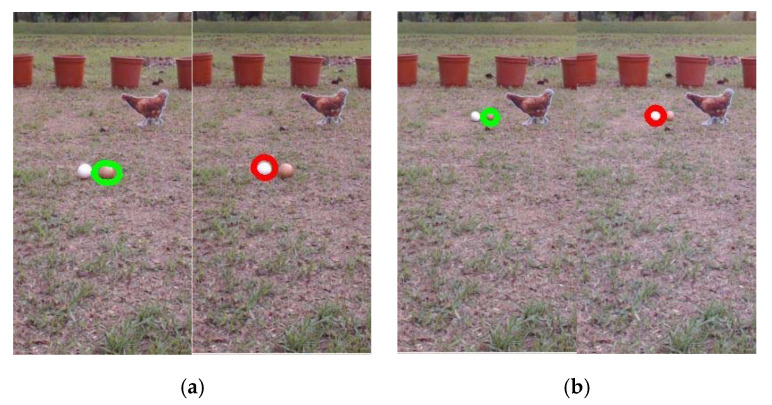
Egg recognition results for different positions: (**a**) 85 cm; (**b**) 105 cm.

**Figure 10:**
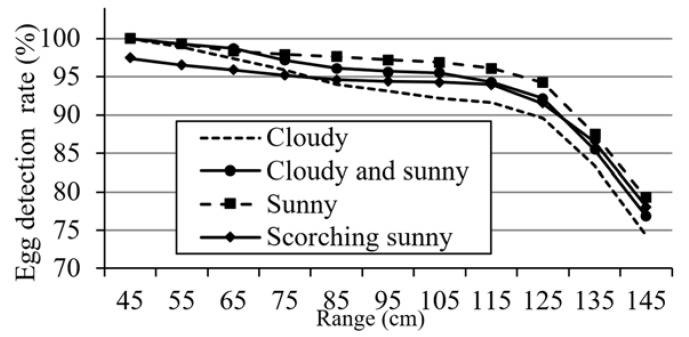
Egg detection results for different distances between robot and egg.

The effect of the vision system on the performance is evaluated in terms of the recognition of eggs at different positions in the image plane. The robot is set at different positions in the field, and white and brown eggs are located in front of it at different distances. The egg detection results obtained at different locations are averaged, and experiments are conducted under three different climatic conditions. Figure 9a,b shows results for the detection of white and brown eggs via the vision system, when the eggs are located 85 and 105 cm in front of the robot under sunny conditions. Figure 10 shows the results for the detection of eggs placed at different distances in front of the robot under different climatic conditions. It can be seen from the figure that the detection rate of eggs within 75 cm from the front of the robot is at least 95%. When the distance is greater than 125 cm, the detection rate is less than 90%. As the range gradually increases, the detection probability drops sharply. The figure also shows that the egg detection rate under sunny conditions is higher than that under other climatic conditions. Under the scorching sunny and cloudy weather, the egg detection rate is lower than that under other weather conditions. As the position of the egg is farther away from the robot, the area of the egg in the image frame is smaller and the egg detection rate decreases.

**Figure 11:**
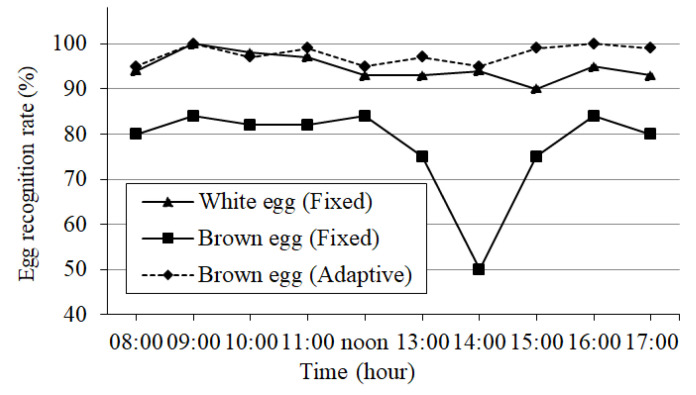
Egg detection rates over different time periods.

Next, white and brown eggs are placed at a distance of 75 cm in front of the robot. The vision system performs egg detection every 10 min, and averages six detection results per hour. The experiment is carried out between 8 a.m. and 5 p.m. every day, for a total of five days. The average detection results for white and brown eggs are shown in Figure 11. It can be seen that when the adaptive thresholding method is used, the average detection rate of brown eggs is 97.6%, while the detection rate for the fixed threshold is only 77.6%. The average detection rate for white eggs is 94.7%. Regarding the recognition of brown eggs, the color threshold under sunny conditions is used. At about two o’clock in the afternoon, the weather changes from sunny to cloudy, which also causes the recognition rate to drop. In contrast, after using an adaptive color threshold, its recognition rate can be increased to about 95%.

### 4.3. Experimental Setup

**Figure 12:**
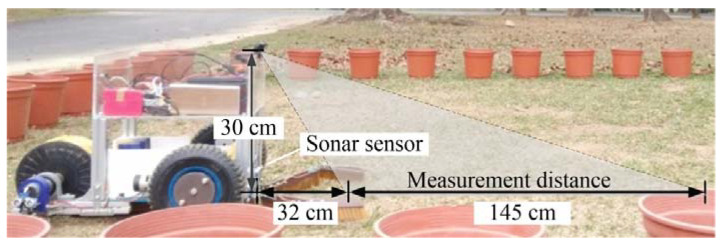
Determination of the visual and ultrasonic detection range for the robot.

The experiment is carried out in an open field at National Pingtung University of Science and Technology (longitude: 120°60′61.30″ E, latitude: 22°64′65.96″ N). The size of the field is 5 m × 5 m, and flower pots are placed around the field. The maximum measurement distance for egg detection is 145 cm (see Figure 12). The initial heading angle and the angle of the field of view are $\theta_{0}=0^{\circ }$ and $\alpha =140^{\circ }$, respectively. During the egg recognition process, the image frame captured by the onboard camera is 640 × 480 pixels (length × width). The obstacle detection distance is set to 32 cm. The speed of the robot can be obtained using Equation (3). The time consumed by the object detection algorithm is 0.3 ms per frame, the maximum speed of robot is 15.3 cm/s, and the operational speed is 10.7 cm/s. Since the robot cannot move backwards, once the egg is visible in area ❹ of the image shown in Figure 1 and has been recognized, it will pass through the collection channel to be gathered by the picking device inside the robot for color classification.

When color of the egg has been confirmed, the microcontroller (Arduino Nano, Arduino Inc., Boston, MA, USA) instructs the program to drive the sorting device, and then transfers the egg to the storage tank for the corresponding color. When the color classification is complete, the numbers of eggs of each color is recorded in the memory.

### 4.4. Performance Evaluation Results

**Figure 13:**
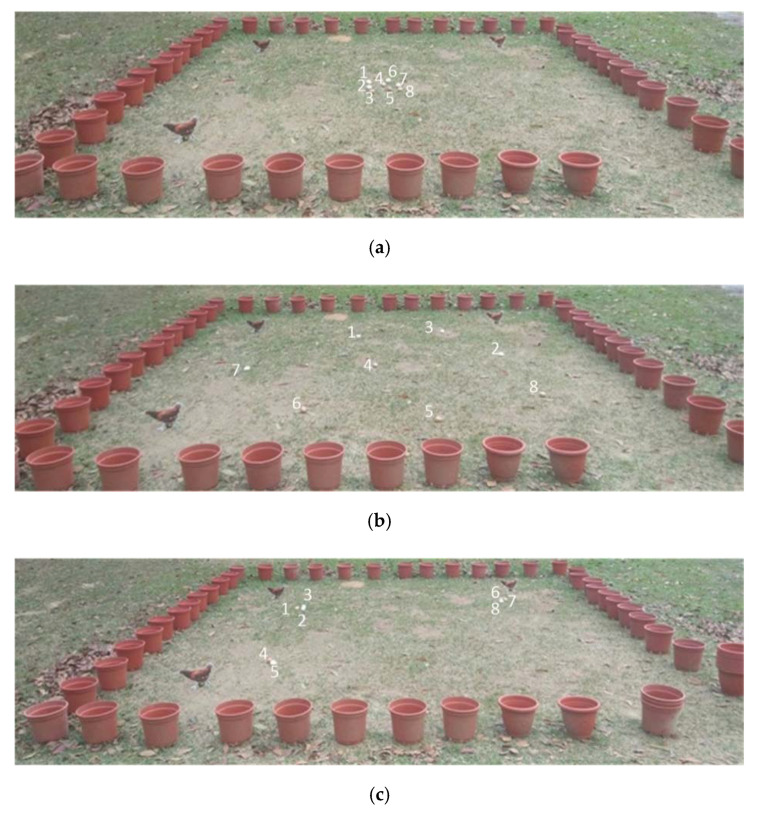
Configurations of eggs in the field: (**a**) central; (**b**) corner; (**c**) dispersed.

In real-life applications, hens will lay eggs at various positions in the field, and most will look for corners or darker areas to lay their eggs. Moreover, laying hens are territorial and will often be near the laying area. In order to evaluate the egg picking performance of the robot under these conditions, eight eggs are placed in three types of configuration: in the center of the field, in the corners, and dispersed across the field, as shown in Figure 13. In the centralized egg configuration, some eggs are close together, and some are slightly separated. There are two types of experiments: with and without obstacles. Three cardboard chickens are used as obstacles, and are fixed at the corners of the field.

**Figure 14:**
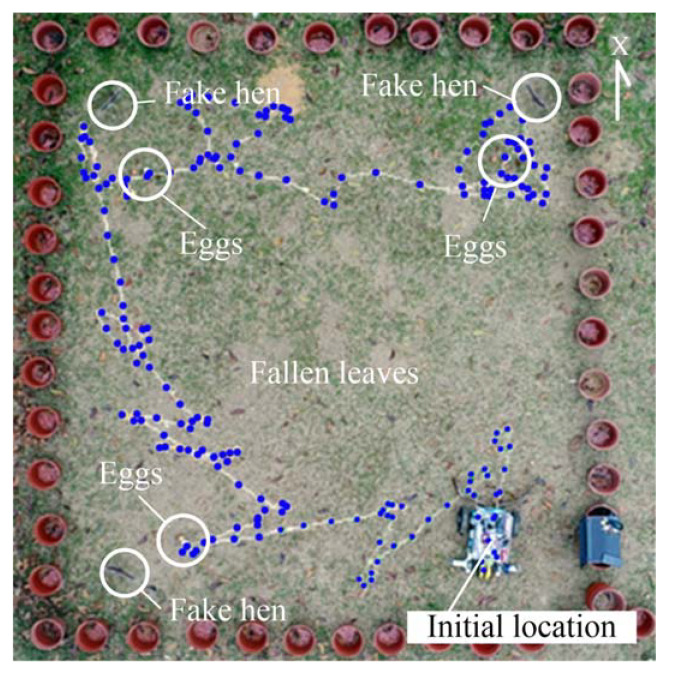
Navigation trajectory for the robot using the GNSS-RTK positioning method.

First, the GNSS-RTK receiver device is used to record the trajectory of the robot, and to investigate its behavior. The climatic conditions on the day of the experiment are sunny. Figure 14 illustrates the robot navigation trajectory obtained by the GNSS-RTK positioning method, where the blue points indicate the positioning point of robot movement. The figure shows that the robot can correctly avoid the cardboard hens and continuously carry out egg detection in the field. However, it can also be seen from the figure that small objects far away from the robot, such as fallen leaves, can cause the vision system to misclassify them as eggs, causing the robot to move toward them. Since there are ultrasonic sensors on both sides of the robot’s chassis, it can avoid collisions with the flower pots when it rotates.

**Figure 15:**
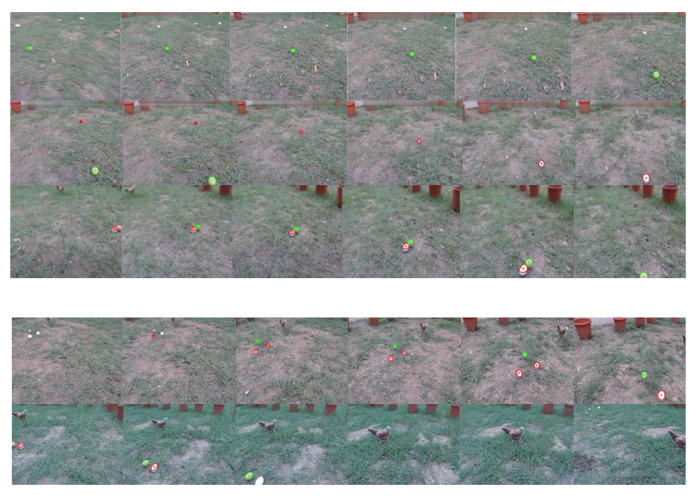
Snapshot of the movement of the robot: egg tracking (**top**) and avoidance of the cardboard chickens used as obstacles (**bottom**).

The real-time egg detection results using the vision system are shown in Figure 15. It can be seen that the features of the eggs can be continuously captured, and that the robot moves in the direction of each egg. The lower image in Figure 15 shows that the robot can avoid obstacles in real time. After that, six experimental results are analyzed, with and without obstacles and with different types of configuration of the eggs. Each egg collection experiment is repeated six times and the experimental results are averaged. Table 3 shows the average egg-picking times for different egg configurations. The definition of the time required for manual egg picking includes the time to find the egg, the time to move to the egg, and the time to pick it up. The walking speed of young people is about 1 m/s. Each experiment is repeated six times, and the results are averaged. It can be seen that both with and without obstacles, the dispersed configuration requires the longest collection time, followed by the corner and central configurations. Manual egg collection requires the shortest time: in the best case, all of the eggs in the field can be picked up within 15 s.

**Figure 16:**
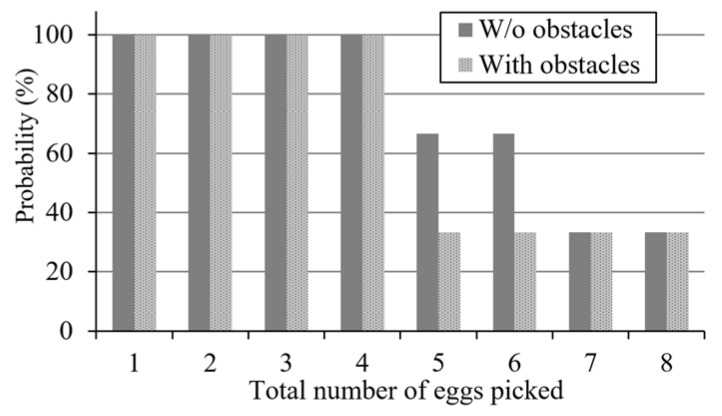
Probability of egg collection within a limited collection time of 5 min.

Next, the egg collection time is limited to five minutes, in order to evaluate the performance of the robot. The results of each experiment show that the robot can collect at least four eggs from the field with a probability of 100%. The probability of collecting more than seven eggs is only 34%. The results are shown in Figure 16. It is worth noting that when there are no obstacles in the field, the probability that the robot can collect 5–6 eggs in a limited time is about 33% higher than that in the case of obstacles. It can be observed that the presence of obstacles will reduce the efficiency of egg collection by the robot.

**Figure 17:**
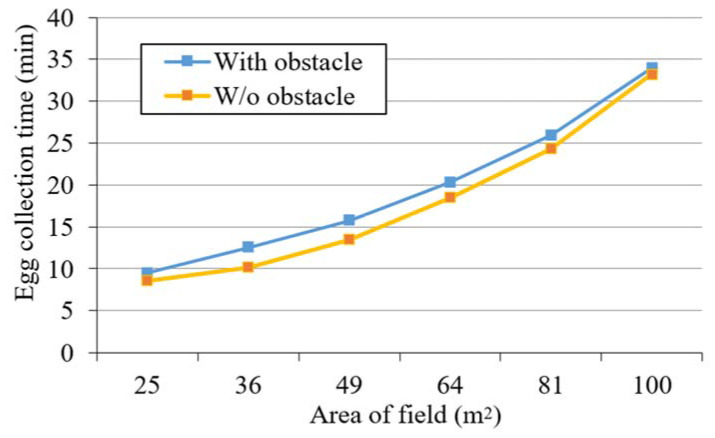
Size of the field versus egg collection time.

Finally, the area of the field used in the experiment gradually increases from 25 to 100 m^2^. The types of configuration used for the eggs, the numbers of eggs, and the number and distribution of obstacles are the same as those described above. Figure 17 shows the results in terms of the time needed for the proposed robot to collect eggs from fields of different sizes, for the decentralized configuration with eight eggs. It can be seen that as the area of the field increases, the egg collection time increases nonlinearly. In addition, it is also observed that when the area of the field increases to more than 81 m^2^, the egg collection time is similar with and without obstacles.

### 4.5. Discussion

Our mobile poultry robot is suitable for most flat feeding grounds, and can be applied to chicken farms with a small area. It takes less time to pick up eggs manually than to use robots to pick up eggs, and manual picking also requires labor in terms of moving around. For larger fields, more labor and time are required. It is therefore feasible and effective to use robots for egg-picking operations. A stone that is similar in size to an egg may block the passage. However, as the robot rotates, this can be avoided. It is worth noting that the vision system will incorrectly judge objects with a similar brown color and size (such as fallen leaves in a small area) as brown eggs, and will move in the direction of these objects. It is, therefore, necessary to remove these objects from the field. However, this also requires additional manpower. Therefore, this system is more suitable for use in spring or fields with no fallen leaves.

In the initial study, the robot uses a spiral path to collect eggs using a wall-following navigation method. The experimental results show that the robot is able to collect eggs from flat indoor ground, with an egg picking rate of 60%–88%. However, when the size of the field becomes larger and the terrain is uneven, the navigation path of the robot will deviate from the desired path. The longer the path along which the robot moves, the more position error will increase, resulting in insufficient coverage of the collection area. A behavior-based navigation method is then applied to allow the mobile robot to detect eggs in an outdoor field, with an arbitrary path. When eight eggs are placed in the field, the robot took about 10 min to gather all of them. At present, the robot can be successfully applied to a field size of 100 m^2^. If this robot needs to be used in a large field, it is recommended to divide the large field into multiple small fields and use multiple robots to complete the work of picking eggs. It is also possible to enlarge the size of the robot and increase the battery capacity. Of course, this robot is also suitable for collecting eggs when people are raising poultry in the backyard.

On the other hand, the cardboard chicken used in the experiment is placed in the corner of the field. In a wide chicken farm, the actual chickens should keep a certain distance from the robot when they see the robot moving. However, if the hens are hatching eggs, the chickens may not leave the laying area. At this point, the robot should be able to avoid the hens. This may be confirmed in subsequent research work.

This study presents an image-processing technology with adaptive threshold regulation, which can improve the detection rate of eggs. The experimental results show that this method allows the robot to recognize eggs under fluctuating lighting conditions, and its detection rate can reach up to 97.6%. In addition, the use of this egg collection system can reduce egg collisions or cracks caused by pressure. Our design can be extended, with multiple collection systems used to collect a large number of eggs quickly.

The contributions of this study can be summarized as follows:

- A combination of color and shape are extracted as features for use in the image-processing method for object detection. It is suitable for detecting small objects in the environment, such as hen’s eggs on the ground or duck eggs;
- The use of automatic thresholding can reduce the effect of light intensity on egg recognition;
- The modular design of the mechanism, which includes an egg collection channel, an egg-picking and -sorting mechanism, and a storage tank, can be easily expanded into a large-scale platform to store larger numbers of eggs;
- A behavior-based navigation method based on visual guidance allows the robot to collect all of the eggs on a small free-range farm within a short time.

## 5. Conclusions

A mobile poultry robot with a computer vision-based platform is designed and implemented in this study. A novel mechanism is designed for the robot, including an egg collection channel, an egg-picking turntable mechanism, a sorting device, and a storage tank, to enable it to collect, sort, and store eggs. Using our vision system, the eggs can be successfully identified in real time under different climatic conditions in an outdoor environment. Within a visual distance of 75 cm, the egg detection rate can reach 97.6%, and a centralized egg distribution is shown to give the shortest collection time. In addition, the combination of the vision system and behavioral logic control method can effectively allow the robot to avoid obstacles. It is able to gather eight eggs in 10 min, within a field area of 25 m^2^. Since the time needed to collect eggs will increase with the size of the field, farmers can divide a large field into multiple smaller areas and use several robots to collect eggs. Furthermore, combining robots with cyber-physical systems (CPS) and Internet of Things technology can achieve the goal of intelligent egg production management [56].

## Figures and Tables

**Table 1:** Component specifications for the poultry robot.

Description	Value or Feature
**Body**	
Size: length (cm) × width (cm) × height (cm)	60 × 30 × 40
Maximum weight	23 kg
Chassis frame	Aluminum/plastic/acrylic
**Drive components**	
Drive method	Two-wheel differential drive
Maximum speed	0.8 ms^−1^
Motors (gear ratio; torque; speed)	24 V, 34.7 W (1:212; 25 kg/cm; 31 rpm)
Egg picking motor (speed)	12 V (7.7 rpm)
Servo motor (torque; speed)	5 V (3.3 kg/cm; 62.5 rpm)
Battery	12 V, 7.2 Ah
**Electronics**	
Controller boards	Arduino UNO; Raspberry Pi 4 model B
Ultrasonic sensors (distance; effectual angle)	5 V (2–400 cm; 15°)
Light sensors (range; accuracy; communication)	2.4 to 3.6 V (1–65,535 lux; ±20%; I2C bus)
Camera 1	Full HD 1080 p, 30 fps, USB 2.0, AUTO Focus
Camera 2 (pixel count; lens; view angle)	2592 × 1944 (5 megapixel; 3.57 mm; 65°)
GNSS-RTK	ZED-F9P RTK GNSS receiver board

**Table 2:** Relationship between color threshold and light intensity.

Weather Conditions		White Egg			Brown Egg		
	Illuminance (lux)	H	S	V	H	S	V
Cloudy	[0, 3000]	[0, 170]	[0, 30]	[245, 255]	[0, 15]	[75, 135]	[100, 255]
Cloudy and sunny	(3000, 9800]				[0, 200]	[25, 110]	[190, 255]
Sunny	(9800, 35,000]				[0, 10]	[45, 150]	[155, 255]
Scorching sunny	35,001 or more				[0, 12]	[65, 150]	[55, 255]

**Table 3:** Egg collection times (min) for different egg configurations.

	With Obstacles	Without Obstacles	Manual Picking
Corner	8.62	6.61	0.53
Central	4.6	3.62	0.25
Dispersed	9.49	8.53	0.81
